# Influence of Diet on the Bioaccessibility of Active Substances from *Alpinia officinarum* Using In Vitro Digestion Model

**DOI:** 10.3390/molecules30224429

**Published:** 2025-11-16

**Authors:** Wojciech Koch, Justyna Zagórska, Agnieszka Jaworowska, Paweł Jagielski, Ewa Bartusiak-Szcześniak, Wirginia Kukula-Koch

**Affiliations:** 1 Department of Food and Nutrition, Medical University of Lublin, 4a Chodzki Str., 20-093 Lublin, Poland; justyna.zagorska@umlub.edu.pl (J.Z.); agnieszka.jaworowska@umlub.edu.pl (A.J.); 2 Department of Nutrition and Drug Research, Institute of Public Health, Faculty of Health Sciences, Jagiellonian University Medical College, 31-066 Kraków, Poland; paweljan.jagielski@uj.edu.pl; 3 Department of Pharmaceutical Sciences, Collegium Medicum, Jan Kochanowski University in Kielce, IX Wieków Kielc 19a, 25-516 Kielce, Poland; ewa.bartusiak-szczesniak@ujk.edu.pl; 4 Department of Pharmacognosy with Medical Plants Garden, Medical University of Lublin, 1 Chodzki Str., 20-093 Lublin, Poland; virginia.kukula@gmail.com

**Keywords:** *Alpinia officinarum*, bioaccessibility, HPLC, LC-MS, in vitro digestion

## Abstract

*Alpinia officinarum* is a plant widely recognized and utilized in Asian countries as a spice, owing to its diverse aromatic properties and distinctive flavor. In addition to its culinary values, this plant has several valuable pharmacological properties, which have become the subject of research in recent years. Its important biological activities include antibacterial, antioxidant, anti-inflammatory, and anticancer effects. Despite the growing interest in this plant, little is known about the bioaccessibility of its active compounds, which limits the possibility of fully utilizing its therapeutic potential. Consequently, assessing the actual bioaccessibility of pharmacologically active compounds is of paramount importance towards rational use in the prevention and treatment of diseases. This study aimed to analyze the active compound content of the root of *A. officinarum* and evaluate their bioaccessibility through a combination of in vitro digestion methods utilizing cellulose membranes, alongside HPLC and HPLC-MS analyses. Furthermore, the impact of the food matrix on bioaccessibility parameters was also examined. The results identified twelve major constituents within the root, with galangin at the highest concentration. Across all dietary models, galangin exhibited the highest bioaccessibility parameter (17.36–36.13%). The variability in results for specific compounds suggested a significant influence of the food matrix on their bioaccessibility. Thus, it can be concluded that the dietary matrix plays a crucial role in modulating the bioaccessibility of active compounds derived from *Alpinia* root, contingent upon the molecular type and composition of the respective diet.

## 1. Introduction

In recent years, there has been a notable increase in the number of scientific publications focused on the investigation of plant extracts and their potential applications in pharmacotherapy, attributed to the presence of many bioactive components exhibiting beneficial biological effects [1,2]. The antioxidant capacity and other biological properties of polyphenolic compounds, including their anti-inflammatory effects, render them valuable constituents of a healthy diet [3,4].

*Alpinia officinarum (A. officinarum)* Hance is a member of the Zingiberaceae family, which constitutes the most prominent family within the order Zingiberales. This family comprises 230 species, among which, key representatives include ginger (*Zingiber officinarum*), *A. officinarum*, and turmeric (*Curcuma longa*). Commonly known as galangal, *A. officinarum* is indigenous to parts of Eastern and Southern Asia, particularly Indochina. It is recognized by various local names: ryokyo in Japan, havlıcan and khoulanjan in Turkey and Iran, chitrarathai chooranam and aichhia or dum aidu in India, and gao linang jiang or heha in China [5]. The genus *Alpinia* is one of the most significant members of the Zingiberaceae family. It has been traditionally employed in Asian cultures to prevent and treat numerous ailments, including catarrh, rheumatism, halitosis, ulcers, coughs, throat infections, and gastrointestinal infections [6,7]. Review articles on the phytochemistry, biology, toxicology, and pharmacology of the *Alpinia* genus suggest that the bioactive compounds present in extracts of this plant may modulate critical metabolic pathways and function as natural inhibitors of enzymes implicated in the pathogenesis of chronic diseases such as diabetes, obesity, hypertension, cancer, and inflammation. The rhizomes of *A. officinarum* are particularly rich in flavonoids, phenylalkanoids (such as shogaol and gingerol), diarylheptanoids, and glycosides [8,9,10,11]. All parts of *A. officinarum* (aerial parts, leaves, roots, and rhizomes) are utilized for medicinal purposes, either directly or in the form of extracts [12]. Due to the specific parts of the plant used and the various extraction methods and solvents employed, the composition of the resulting extracts varies, as do their biological activities and therapeutic applications [13]. For instance, a study by Basri et al. identified 16 chemical compounds in the methanol extract of galangal, of which, 12 were flavonoids and 4 were diarylheptanoids (DAHs). Additionally, five flavonoids were characterized from the ethanol extract of the aerial parts of *A. officinarum* [13]. In general, the fresh, dried, and powdered rhizomes and roots of *Alpinia officinarum* are among the most sought-after natural medicines in the retail market, utilized both externally and internally. Their slightly bitter, pungent, and biting flavor—akin to ginger and a sweet and bitter aroma—renders them popular in culinary applications, primarily as spices or natural ingredients for tincture production [14]. Moreover, extracts from this plant are increasingly incorporated into food products as natural flavor enhancers and to boost nutritional value. For instance, a study conducted by Mandal et al. demonstrated that incorporating galangal rhizome significantly enhanced the dish’s nutritional profile, antioxidant activity, and flavor [11,15,16]. The safety of *A. officinarum* for both oral and external use has been substantiated through clinical trials. Administration of an alcoholic extract from the rhizome in rodent models did not indicate any signs of toxicity or mortality within 24 h, nor did it result in alterations to liver or kidney function markers [14,16,17].

The advancement of the market for novel pharmaceuticals derived from bioactive compounds in natural products may encounter significant challenges, particularly regarding the limited bioaccessibility of these compounds [18]. Bioaccessibility is intrinsically linked to digestibility, which refers to the susceptibility of nutrients to digestive enzymes. The bioaccessibility process can be delineated into several stages: the release of the ingredient from the organic matrix in the digestive tract, its passage through the intestinal wall, subsequent metabolism, and eventual entry into systemic circulation. Consequently, research indicates that bioaccessibility of active substances administered in pure form is generally higher than when consumed as part of food [19,20,21]. Various methodologies exist for testing bioaccessibility; however, challenges arise due to the difficulty of obtaining in vivo intestinal contents. Clinical trials are often prolonged, costly, and encumbered by ethical considerations, yet they validate research hypotheses. This challenge has prompted the development of digestive models and in vitro systems. These models facilitate rapid and controlled assessments of bioaccessibility, serving as invaluable tools for screening studies and hypothesis formulation. The processes are conducted under conditions closely mimicking physiological environments, including appropriate pH and fluid volumes. Dialysis systems and intestinal epithelial cell lines serve as models, with the Caco-2 cell line being the most extensively used for investigating the potential absorption of nutrients and other food components. The most sophisticated model available is the Gastro-Intestinal Model (TIM), a computer-monitored Dutch system that simulates peristaltic movements, pH regulation, and the incorporation of relevant digestive enzymes [22,23,24,25].

In this study, we employed a two-phase in vitro digestion model utilizing cellulose dialysis membranes to assess the bioaccessibility of the primary active compounds present in the root of *A. officinarum* under varying dietary conditions. We conducted qualitative and quantitative analyses of the active compounds in *Alpinia* root before and after in vitro digestion using liquid chromatography coupled with mass spectrometry (LC-MS/MS) and high-performance liquid chromatography (HPLC) techniques. This analysis aimed to calculate the bioaccessibility parameter for each compound and model utilized in the study, with an additional objective of determining the composition of the extract from *A. officinarum* rhizome employed in the experiments.

## 2. Results

### 2.1. HPLC-MS Fingerprinting of Alpinia officinarum Extract

The gradient settings used provided suitable conditions for the effective separation of metabolites present in the total extract.

**Figure 1:**
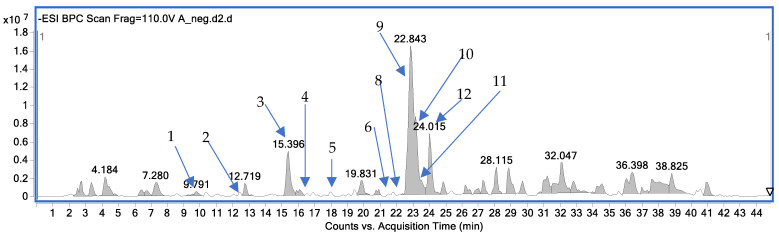
Composition of the *A. officinarum* extract with the identified compounds from the LC-MS analysis in the negative ion mode (1-zingerone, 2-3-phenylpropanoic acid, 3-pinobanksin, 4-kaempferol, 5-1,8-cineole, 6-chrysin, 8-pinocembrin, 9-galangin, 10-kempferide, 11-apigenin, 12-acacetin).

The variety of compounds from different groups of metabolites were tentatively identified in the extract from *Alpinia officinarum* as the main components in the sample. Table 1 and Figure 1 present the assigned molecules with detailed MS parameters that enabled the tentative identification. The MS/MS spectra of the assigned components are presented in Table S3 in the Supplementary Materials.

The analysis underlines a rich diversity of bioactive compounds within the studied species, predominantly within the flavonoid and phenolic acid groups. The observed fragmentation patterns not only prove the structural identities of these metabolites but also suggest potential pathways for their metabolic processing within biological systems.

Among the key assigned metabolites, galangal, flavonoids (kaempferol, galangin, kaempferide, chrysin, apigenin, isorhamnetin), phenolic acids (3-phenylpropanoic acid), and terpenoids (zingerone, 1,8-cineole) were found in this study.

The determined MS/MS fragmentation patterns provided additional insights into the structural characteristics of the identified compounds. In the case of the largest peak of galangin (peak 9), two strong fragments at 227.0360 and 213.0564 Da were observed, which indicated the loss of CO_2_, or CO and water moieties during the analysis.

The fragmentation of kaempferide speaks for its structural similarity with kaempferol owing to the presence of the 284.0318 ion. In the case of apigenin—the third most intensive compound—a typical fragment of 227.0343, which comes from the detachment of CO_2_ moiety, was observed.

Figure 1 illustrates the chromatogram recorded in the negative mode of the LC-MS analysis of the *A. officinarum* extract, detailing the composition of low-molecular-weight components (*m*/*z* range 100–1000 Da). The chromatogram displays several prominent peaks, each corresponding to specific bioactive compounds present in the extract.

Among the most significant peaks, peak 9 (galangin) had the highest intensity at 22.843 min, potentially indicating its predominant presence in the extract. Following closely was peak 10 (kaempferide), which exhibits a substantial signal at 24.015 min.

The high abundance of galangin aligns with quantitative findings that are presented below, suggesting its potential as a key therapeutic agent.

### 2.2. HPLC Determinations

HPLC with DAD (Diode Array Detector) detection was employed to conduct quantitative determinations of the active compounds present within the extract of *Alpinia officinarum*. Using a previously validated method with 6-gingerol as a standard, the content of selected active substances in the extract was determined. Galangin (1090.9 mg/kg) was determined to be the primary compound, which was far above the content of the other compounds, followed by kaempferide and apigenin underscoring galangin’s potential as a key specialized metabolite derived from this plant. Following galangin, kaempferide and apigenin were the next most abundant compounds, with respective concentrations of 173.8 mg/kg and 140.0 mg/kg. Notably, the levels of acacetin and chrysin were comparable, with concentrations of 96.1 mg/kg and 98.8 mg/kg, respectively. In contrast, pinobanksin was identified as the least prevalent compound within the powdered rhizome, with a concentration of only 42.0 mg/kg. This amount is particularly striking, as it is 26 times lower than that of galangin, emphasizing the significant disparity in the concentration of these bioactive substances. Table 2 shows the quantitative data of the six major compounds identified in the extract, which were quantitatively determined and for which the bioaccessibility parameters were calculated.

### 2.3. Bioaccessibility Results

The obtained results (Table 3) revealed a significant effect of diet type on the bioaccessibility of particular compounds (*p* ≤ 0.05 for all compounds except acacetin).

The data presented in Table 3 indicate that individual compounds are characterized by markedly different bioaccessibility parameters. These data are graphically represented in the Supplementary Material as Figure S1. For the control sample, corresponding to consumption on an empty stomach, the highest bioaccessibility was observed for acacetin, while the lowest was recorded for apigenin. The results from the control samples suggest that the active compounds in the *A. officinarum* rhizome exhibit significant variability in bioaccessibility, which may subsequently influence their potential biological activity.

The determinations also revealed the food matrix’s pronounced impact on the bioaccessibility of specific compounds. Notably, pinobanksin demonstrated the highest bioaccessibility in the model utilizing a basic diet (16.55%), significantly higher than that observed in the other dietary models. Conversely, the high-fiber diet markedly reduced the bioaccessibility of this compound to 2.30%. Similarly, the presence of the food matrix significantly enhanced the bioaccessibility of apigenin, with the highest value recorded in the standard diet model (40.72%).

For galangin, a gradual increase in bioaccessibility was noted across different dietary types, with the highest value found in the standard diet (36.13%) and the lowest in the control sample (17.36%), which represented a model without food (empty stomach). In contrast, kaempferide exhibited the highest bioaccessibility in the control sample model (8.90%), significantly surpassing all dietary models, while the lowest bioaccessibility was observed in the basic diet model (3.44%).

ANOVA revealed a statistically significant difference (*p* = 0.0309) in bioaccessibility for acacetin, specifically when comparing the high-fiber diet to the basic diet. However, this parameter remained high across all models evaluated in the study. The high-fiber diet also significantly increased chrysin bioaccessibility to 63.60%, whereas the standard diet resulted in a drastic reduction to 3.33%. Chrysin exhibited the greatest variability in bioaccessibility, depending on the experimental model employed.

The most substantial differences between dietary models were observed for apigenin and chrysin, suggesting the potential for strong interactions between these compounds and nutritional components. It was determined that the high-fiber diet could either enhance (for chrysin and apigenin) or diminish (for pinobanksin and kaempferide) bioaccessibility, underscoring the necessity for individual analyses of the influence of nutritional components on bioaccessibility in the context of dietary supplements or functional foods containing *A. officinarum*.

## 3. Discussion

Based on an analysis of the available data from scientific databases, to the best of our knowledge, this study represents the first evaluation of the bioaccessibility of active components derived from the root of *Alpinia officinarum* utilizing an in vitro digestion model with cellulose membranes across various dietary conditions. The existing literature predominantly contains studies on the composition of the plant that showed the presence of phenolic compounds in its extracts [13]. HPLC-MS analysis facilitated the identification of 12 distinct compounds in *A. officinarum* root, including pinocembrin, chrysin, galangin, acacetin, isorhamnetin, kaempferide, pinobanksin, zingerone, 3-phenylpropanoic acid, apigenin, kaempferol, and 1,8-cineole. Among these, six compounds—pinobanksin, apigenin, galangin, kaempferide, acacetin, and chrysin—were selected for further bioaccessibility assessment. Their presence was tentatively assigned using mass spectrometry and high-resolution *m*/*z* measurements, double bond counts, fragmentation profiles at a collision energy of 20 V, and with respect to an abundance of scientific literature describing the composition of galangal extracts. A previous study by Patir et al. employing HPLC-DAD identified significant peaks corresponding to galangin, quercetin, kaempferol, isorhamnetin, and chrysin [26]. Conversely, Jiao et al. reported that extracts from *A. officinarum* rhizomes contained phenolic compounds such as galangin, galangin-3-methyl ether, pinobanksin, and kaempferol-4-methyl ether [27]. The findings suggest that the investigated root, akin to *Zingiber officinale*, is abundant in phenolic compounds with notable medicinal properties, particularly antioxidant activity [28].

Moreover, the content of selected active substances in the root was quantified using a previously optimized and validated method [29]. Notably, galangin exhibited a significantly higher concentration than other active substances, while pinobanksin showed the lowest concentration. Previous quantitative analyses of *Alpinia* rhizome reported elevated levels of pinobanksin, alongside high concentrations of galangin and zingerone [29]. These observations indicate that, while the qualitative composition of the rhizome and root may be similar, the concentrations of individual compounds can vary considerably, influenced by factors such as storage, processing, and the freshness of the plant material [30].

The digestion and filtration studies utilizing cellulose membranes enabled the determination of the bioaccessibility of selected compounds across various dietary models: control (without dietary matrix), high-fiber, standard, and basic diets. The results revealed significant bioaccessibility variability, attributable to differences among individual compounds and the impact of the food matrix. The high-fiber diet, characterized by a fiber content twice that of the other diets and a substantial concentration of vitamin C, emerged as particularly distinctive. The basic and standard diets demonstrated similar energy values and lipid contents; however, the standard diet contained markedly higher levels of protein and calcium, whereas the basic diet was enriched in carbohydrates and exhibited elevated levels of dietary fiber, and vitamins C and E. These findings underscore the substantial influence of dietary matrices on the bioaccessibility of the analyzed active compounds post-in vitro digestion, which directly correlated with the nutritional composition of each respective diet. Statistically significant differences (*p* < 0.0001) were observed between sample groups, confirming the critical role of the dietary matrix in models of simulated gastrointestinal digestion. For pinobanksin, the highest bioaccessibility was recorded in the basic diet model (16.55%), markedly exceeding that in the high-fiber diet (2.30%), suggesting that dietary fiber may inhibit the availability of this compound. To date, there is no definitive scientific evidence regarding the interaction of antioxidants with other food components, such as dietary fiber. However, it is known that fiber can limit the bioavailability of macronutrients, particularly fats and some minerals and trace elements, in the human diet. In addition, pectin has been shown to significantly reduce the bioavailability of β-carotene, suggesting that dietary fiber may also affect the absorption of other carotenoids, as well as α-tocopherol compounds and polyphenols. In general, dietary fiber may have two main functions in the intestine: prolonging gastric emptying time and delaying nutrient absorption. Both are dependent on the physicochemical form of the fiber, particularly its effect on chyme viscosity [31,32]. Conversely, chrysin exhibited a bioaccessibility of 63.6% in the high-fiber diet model, significantly surpassing the other models, including the control, indicating that dietary fiber may enhance the bioaccessibility of this compound. Notably, chrysin is recognized for its low oral bioavailability due to poor solubility and moderate permeability [33,34,35]. Ongoing research is focused on enhancing chrysin’s bioavailability through innovative approaches such as nanocarriers or conjugated micelles to optimize its therapeutic potential [36,37]. The results of the current study may imply that dietary fiber could exert a protective effect by forming complexes that shield the compound from enzymatic degradation. Nonetheless, further in vivo and clinical investigations are essential to validate this hypothesis.

A high bioaccessibility was noted for apigenin in the standard diet model (40.72%) and the high-fiber diet (20.57%), whereas the control and basic diet samples exhibited significantly lower values. Interestingly, the high-fiber diet markedly improved apigenin availability. In vitro and in vivo studies have reported rapid metabolic rates and low bioavailability of apigenin, emphasizing the importance of selecting appropriate doses to achieve desired clinical outcomes [38]. Numerous investigations have sought to enhance apigenin’s bioavailability due to its inherently low oral bioavailability. The flavonoid is estimated to have solubility rates of approximately 0.001–1.63 mg/mL in nonpolar solvents and around 2.16 μg/mL in aqueous solutions, suggesting that the dietary composition can significantly affect solubility and, consequently, bioaccessibility [39,40]. The substantial variability in apigenin’s solubility may explain the pronounced differences observed in its bioaccessibility, which is dependent on the dietary composition or the absence of food. Furthermore, the findings of this study indicate that judicious meal selection may significantly enhance the bioaccessibility of this compound.

Amidst the rising incidence of cancer in recent decades, galangin and extracts rich in this compound are garnering increasing attention due to their potent anticancer properties [41,42,43]. This highlights the imperative to investigate the bioaccessibility of galangin and other constituents within galangal, as enhanced bioavailability could augment the therapeutic efficacy of these compounds. Current research has demonstrated moderate to high bioaccessibility for galangin, which is strictly dependent upon the dietary matrix employed within the specific models. Relative to other compounds assessed in this study, galangin exhibited satisfactory bioaccessibility across all dietary models and control samples, establishing it as the most bioavailable compound evaluated. The highest bioaccessibility was observed in conjunction with the standard diet (36.13%), with promising results also evident in the basic diet model (23.64%). The high-fiber diet yielded relatively favorable results, suggesting no significant constraints from the dietary fiber component. The number and positioning of hydroxyl groups within the galangin molecule are known to profoundly influence its biological efficacy; however, in vitro studies indicate that these molecules undergo glucuronidation and sulfation. Nevertheless, systemic exposure to galangin post-administration in animal or human models remains mostly unexamined, raising uncertainties regarding whether galangin achieves sufficiently high concentrations within the body to exert biological effects [44]. In a study by Curti et al., the bioavailability of galangin, among other key polyphenols in brown propolis extract, was evaluated in murine models. However, neither galangin nor its derivatives were detectable in the bloodstream, precluding a comprehensive bioavailability analysis. Oral administration of the extract was observed to facilitate rapid absorption and metabolism of galangin, subsequently activating the first-line antioxidant defense mechanisms [45].

Kaempferide exhibited the lowest bioaccessibility among all the compounds evaluated in this study. The lowest values were determined in the basic diet model (3.44%), with the highest values noted in the control model (8.90%), suggesting that the food matrix significantly impairs the availability of this compound. A review of existing literature did not reveal challenges regarding the bioavailability of kaempferide, which is categorized as a flavonoid possessing substantial biological activity, with numerous studies dedicated to its anticancer properties. However, these investigations did not encompass nutritional matrix considerations [46,47,48,49,50,51]. Research by Walle and Walle indicated that of the 15 methoxylated flavones they assessed, two partially methylated compounds, tectochrysin and kaempferide, demonstrated heightened susceptibility to microsomal oxidation [52]. Furthermore, in studies conducted using the 3T3-L1 cell model, kaempferide exhibited the highest cellular uptake coefficient among the tested compounds, alongside the most pronounced inhibitory effects on adipogenesis [53]. However, elevated cellular uptake does not necessarily correlate with high bioaccessibility, as the latter is a multifaceted process influenced by various factors. Consequently, results may vary considerably based on the employed model or experimental conditions.

The variability among samples was less pronounced for acacetin, though some differences were statistically significant. Overall, this compound exhibited high bioaccessibility, with the highest values observed in the basic diet model, indicating a moderate positive influence of the food matrix on its bioaccessibility, albeit with insignificant differences compared to the control. A study by Han et al. established that acacetin is characterized by poor solubility and relatively low stability within a pH 7 phosphate buffer and simulated gastrointestinal fluids. A significant proportion of acacetin remained unabsorbed in jejunal segments, and its bioavailability following oral administration in rats was notably low [54]. Conversely, investigations into the bioaccessibility of acacetin from Korean mint (*Agastache rugosa*) revealed that rosmarinic acid, acacetin, and tilianin were detectable in plasma following a single ingestion. Acacetin and tilianin exhibited consistent transport across the Caco-2 cell monolayer, indicative of transepithelial transport. Acacetin glycosides with acetyl and malonyl groups were infrequently detected in plasma, and the presence of acacetin glycosides with only an acetyl group on the basolateral side suggests hydrolysis of malonyl glycosides within the large intestine [55].

The results confirm that dietary composition, due to the complexity of the food matrix, can significantly modulate the bioaccessibility of polyphenolic compounds. This factor must be considered when designing pharmaceuticals and dietary supplements containing plant extracts. In vitro digestion models, such as the one utilized in this study, serve as vital and predictive tools for assessing the potential bioaccessibility of active ingredients before conducting in vivo studies. Similar models have been employed to evaluate the bioaccessibility of phenolic compounds in the research undertaken by Sęczyk et al. [28]. Their study indicated that the digestion process, hydrothermal treatment, and food matrix significantly adversely affected compound bioaccessibility, correlating with several findings from the current investigation. The degree of impact observed in Sęczyk et al.’s study was contingent upon the specific phenolic compound, the nature of the food matrix, and the food processing method. Furthermore, their study proposed dose adjustments to compensate for the influence of test factors on the measured properties, presenting an intriguing perspective on the bioaccessibility of phenolic compounds. In research by Zagórska et al., the active substances from *Zingiber officinale* were analyzed, revealing that the digestion process led to a significant reduction in the content of active compounds. Additionally, in a model utilizing a high-fiber diet, the highest bioavailability of active compounds from ginger was observed, paralleling the results of this study and suggesting a protective role of polyphenols against oxidative degradation [28,30]. However, further studies are warranted to assess the bioaccessibility of active compounds from plants within the Zingiberaceae family, including the influence of dietary matrices, to elucidate the complex interactions between active substances and food components.

This study’s strength lies in applying a previously developed and validated method founded on advanced analytical techniques such as LC-MS and HPLC, which enabled the precise quantification of the studied compounds and facilitated accurate calculations of the bioaccessibility parameters for each active component. The results obtained were subjected to rigorous statistical analysis. An additional critical aspect was the formulation and reconstruction of average food rations by a certified dietitian, representing typical dietary patterns within the population. This approach allowed for the simulation of real-world dietary contexts in which *Alpinia* and its derivatives will likely be consumed. It is essential to acknowledge the limitations of this study. It is widely recognized that no singular, ideal method exists for assessing bioaccessibility; each method has inherent limitations. Nevertheless, in vitro techniques, particularly those employing cellulose dialysis membranes, are regarded as among the most effective, cost-efficient, and ethically unobjectionable methods for initially estimating the bioaccessibility of various substances, including drugs, natural products, and components of dietary supplements [56,57,58]. Furthermore, the objective of this study was not to analyze the effects of individual nutrients on the bioaccessibility of active components from *Alpinia*, but rather to analytically assess this parameter and illustrate that dietary type, and consequently the intricate food matrix, can significantly influence the bioaccessibility of the active ingredients in this plant, potentially affecting its biological effects. It is crucial to recognize that *Alpinia* and its preparations can be consumed in diverse forms and manners—as fresh or processed roots, dietary supplements, or functional foods, in conjunction with various foods, or on an empty stomach. Therefore, achieving a complete and precise reproduction of potential interactions through the in vitro model, based solely on analytical determinations, is inherently challenging. Consequently, rather than utilizing pure, isolated nutrients or sample meals, comprehensive, reconstructed daily food rations were employed to illustrate the potential impact of dietary composition on the bioaccessibility of active components derived from *Alpinia* roots.

It is important to note that this study did not specifically address the effects of individual nutrients on bioaccessibility; rather, the results obtained can be regarded as preliminary findings that will inform future investigations into the influence of specific food components on the bioaccessibility of active substances from *Alpinia officinarum* root. This line of inquiry may also extend to other plant species, as our results indicate that food significantly impacts the bioaccessibility of active components derived from natural products, which is likely to affect their overall biological activity.

Considering that oral ingestion is the predominant route of administration for natural products, the influence of digestive processes and the presence of food on their bioaccessibility may bear greater practical significance than merely the concentration of active compounds in the raw material, which is often the primary focus of scientific research. Therefore, further studies are warranted to elucidate the impact of specific food components on the bioaccessibility of active substances present in the root of *Alpinia officinarum* and other natural products.

## 4. Materials and Methods

### 4.1. Plant Material

The research material was the root of *A. officinarum* from wild cultivation in China, which was purchased in March 2021 from the NatVita company (Bojano, Poland) (batch number 43185). The raw material was originally finely powdered, and the package contained 80.328 g of plant powder.

### 4.2. Chemicals and Reagents

*A. officinarum* extract was prepared using 96% ethanol (Avantor Chemicals, Gliwice, Poland) as described in the following Section 2.3. Ultrapure water with a resistivity of 18.2 MΩ•cm was obtained using the Ultrapure Millipore Direct-Q-R 3UV system (Millipore, Bedford, MA, USA). In vitro digestion was performed using NaCl, HCl, and NaHCO_3_ (Avantor Chemicals, Gliwice, Poland), pepsin (≥500 U/mg), pancreatin from porcine pancreas, and porcine bile extract (Sigma-Aldrich, St. Louis, MO, USA). 6-Gingerol (≥98% HPLC, Sigma-Aldrich, St. Louise, MO, USA) was used as a standard for quantitative analysis. Water, acetonitrile (ACN), and formic acid (Merck, Darmstadt, Germany) were used in HPLC-MS analyses.

### 4.3. Preparation of Alpinia officinarum Extract

The extract was prepared following a previously described procedure [29,59]. The powdered root of *Alpinia officinarum* was precisely weighed to an initial mass of 80.328 g and divided into eight conical flasks, each containing 10 g of raw material. Subsequently, 50 mL of 96% ethanol (*v*/*v*) was added to each flask, ensuring complete immersion of the raw material. The flasks were then placed in an Ultrasound Efficiency Emmi 55HC-Q ultrasonic shaker (EMAG Technologies, Mörfelden-Walldorf, Germany), where extraction was conducted for 30 min at a temperature of 30 °C.

Upon completion of this extraction period, the contents of each flask were filtered through filter paper into clean Erlenmeyer flasks. To the residual raw material remaining in the flasks, along with the filter paper, an additional 50 mL of 96% ethanol was added. The flasks were sealed and subjected to ultrasonic extraction for another 30 min at the same temperature of 30 °C. Following the second extraction, the samples were filtered similar to the first extraction .

The contents of half of the flasks were quantitatively transferred to a large round-bottomed flask with a capacity of 1000 mL, after which, the solvent was gradually evaporated using a rotary vacuum evaporator (INGOS RVO400, INGOS, Prague, Czech Republic). The evaporation process was conducted under the following conditions: temperature range of 32–37 °C and a minimum pressure of 100 hPa, utilizing ethanol as the extractant. The samples were concentrated in the vacuum evaporator until approximately half of the extract volume had evaporated. The second portion of the extract was then added, and the evaporation process continued until the concentrate could be transferred to a previously weighed, smaller round-bottomed flask, where it was further evaporated to dryness.

**Figure 2:**
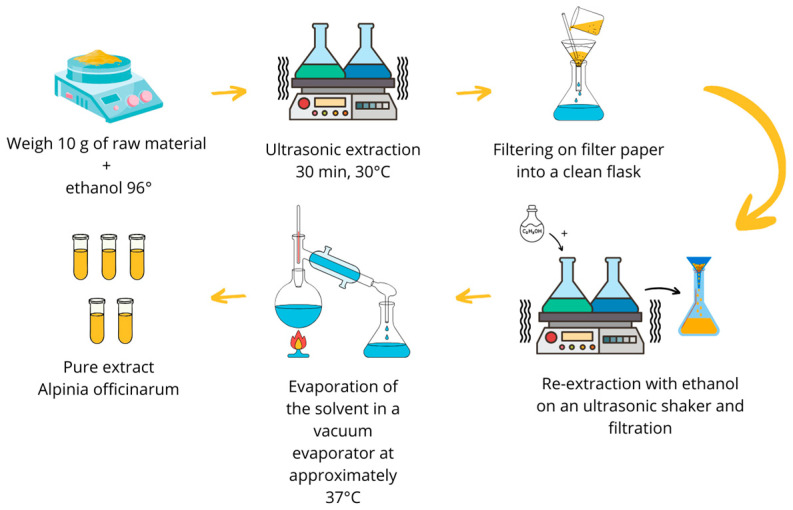
Experimental design of *A. officinarum* extract preparation.

After cooling, the flask was weighed, yielding a dry extract mass of 5.1352 g, resulting in an extraction efficiency of 6.42%. The extract obtained was designated for subsequent studies. The complete process of plant extract preparation is illustrated in Figure 2.

### 4.4. Diet Composition

Three diet models were used to study the bioaccessibility of active components from *A. officinarum*. A high-fiber diet, a standard diet, and a basic diet were used. Their nutritional value was calculated using Dieta 6.0 software (Food and Nutrition Institute, Warsaw, Poland). A detailed description of the development and preparation of the diets can be found in previous works [60]. Briefly, Dietetyk 2006 (Jumar, Poland) and Dieta 6.0 software were used to plan and develop the average daily food rations representing major, common types of human nutrition. The products used to reconstruct the diets were sourced from the local Lublin region. The raw materials were removed from inedible parts, homogenized using a titanium blade, packaged in plastic containers and stored at −20 °C until analysis. The composition and nutritional value of all diets were presented in the Supplementary Materials in Tables S1 and S2.

### 4.5. In Vitro Digestion

The digestion process was started by weighing 0.2 g of the extract into laboratory vessels. Then, 2 g of homogenized diets and 9.6 mL of a mixture containing 20 mg of NaCl dissolved in 0.01 mol/L HCl solution were added to each sample. The whole was mixed thoroughly, and then the pH of the samples was adjusted to 2 using 0.1 mol/L HCl solution. After reaching the appropriate acidity level, 0.4 mL of pepsin solution prepared in 0.1 mol/L HCl was added to each sample. Then, the tightly closed containers were placed in a water bath (Vibra, AJL Electronic, Krakow, Poland) and shaken for 2 h at 37 °C. This stage of digestion corresponds to the processes occurring in the stomach and reflects the acidic environment characteristic of this part of the gastrointestinal tract. The next stage involved neutralizing the samples with 1 mol/L NaHCO_3_ solution until a pH of 6.5 was reached. After determining the appropriate pH, 5 mL of a 4% pancreatin solution with added bile salts (2.5% *w*/*v*), prepared in 0.1 mol/L NaHCO_3_, was added. The sealed samples were placed in a water bath and shaken for 2 h at 37 °C. This part of the study corresponded to the stage of intestinal digestion, in which the environment adopts higher pH values.

To stop the enzymatic processes, 5 mL of methanol was added to each sample and then centrifuged for 10 min at 3000 rpm (Steinberg Systems centrifuge, Berlin, Germany). After centrifugation, the volume of each sample was supplemented with ultrapure water to a volume of 40 mL.

Half of the volume of the obtained samples was prepared for HPLC analysis, for which purpose the material was filtered through a syringe filter. The other half of the samples was prepared for filling dialysis membranes. The membranes used for the study were previously soaked for 12 h in 0.1 mol/L HCl and rinsed with ultrapure water before use. The tested solution of 20 mL was poured into a dialysis membrane with a molecular weight cut-off (MWCO) of 14 kDa (Dialysis Tubing, Benzylated, Sigma-Aldrich, St. Louis, MO, USA) and tightly closed with a laboratory clip (Sigma-Aldrich, St. Louis, MO, USA). The membranes were placed in a laboratory container with a screw cap, filled with ultrapure water (V = 200 mL), and shaken in a water bath for 2 h at 37 °C. The fluid volumes were measured after the filtration process was completed, and the values were included in further bioaccessibility calculations. The remaining fluid inside the membrane was filtered through a syringe filter (Cronus Filters, Gloucester, UK, pore size Ø 0.22 μm) for further analysis.

**Figure 3:**
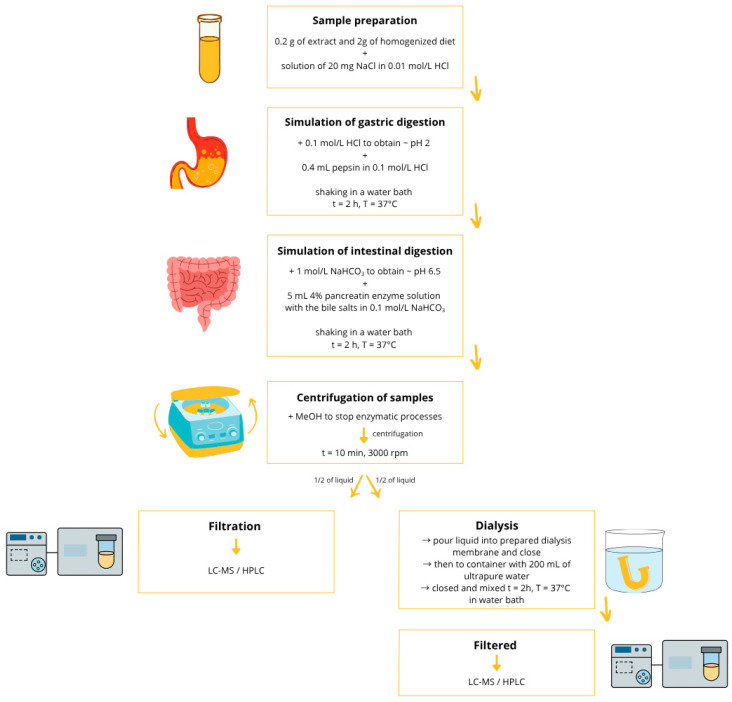
In vitro digestion process.

Ultrapure water was added to the samples instead of the diets for the control tests. In addition, enzymatic control tests were performed without extract and without dietary models. The remaining steps of the procedure and the reagents used were identical to those in the test samples. All determinations were performed in triplicate. The method for model digestion and bioaccessibility determination was developed and optimized in previous works [30,60]. The entire in vitro digestion protocol is shown in Figure 3.

### 4.6. Qualitative Analysis Using the LC-MS Method

High-Performance Liquid Chromatography-Electrospray Ionization-Quadrupole Time-of-Flight Mass Spectrometry (HPLC-ESI-QTOF-MS) from Agilent Technologies (Santa Clara, CA, USA) was used to analyze the composition of the *A. officinarum* extract. An HPLC chromatograph (1200 series) with a Zorbax EclipsePlus RP-18 column (150 mm × 2.61 omfm17; dp = 3.5 μm by Agilent Technologies, Santa Clara, CA, USA) was equipped with a degasser (G1322A), a binary pump (G1312C), an autosampler (G1329B), a diode array detector (DAD) (G1315D), and a mass spectrometer (G6530B). Mass Hunter Workstation software (Agilent Technologies, Santa Clara, CA, USA, version B.10.00) was used to acquire MS spectra and process the data. The extract prepared in the previous steps was dissolved in methanol at a concentration of 10 mg/mL and then filtered through a syringe filter (Cronus filters, pore size Ø 0.22 μm). LC-MS fingerprinting of the samples was used to identify the compounds.

The whole process was as follows: each sample (5 μL) was injected onto the chromatographic column and separated at room temperature using a flow rate of 0.2 mL/min using gradient elution. The whole separation lasted 45 min. The mobile phases contained eluent A (water with 0.1% HCOOH added) and eluent B (ACN solution with 0.1% HCOOH added). The solvent system used in the LC-MS method is given in Table 4.

The applied settings of the mass detector were: *m*/*z* range of 100–1700 Da; gas and sheath gas temperatures of 275 and 325 °C, respectively; gas and sheath gas flows of 12 L/min; fragmentation energy of 110 V; collision energies of 10 and 20 V; capillary voltage of 3000 V; and skimmer voltage of 65 V.

### 4.7. Quantitative Analysis Using HPLC Method

The studied samples for each diet model, before and after filtration through cellulose membranes, were analyzed on a Shimadzu Prominence-i Model LC-2030C 3D Plus HPLC chromatograph (Shimadzu, Kyoto, Japan), and the bioaccessibility of selected compounds was determined in comparison with the standard, which was 6-gingerol at a concentration of 1 mg/mL. Samples in 2 replicates were injected each time in three replicates, and the analysis was performed using the same column, solvents, and gradient elution mode as in the LC-MS determinations. The chromatograph settings were as follows: thermostat temperature of 25.0 °C, flow rate of 0.2 mL/min, injection volume of 5 μL at a concentration of 10 mg/mL, UV detection range of 190–500 nm, and DAD detection wavelength of 290 nm. The 6-gingerol standard solution (1.0 mg/mL in methanol) was used as an external standard for quantitative determinations. Because all identified compounds in the extract represent polyphenol structures and behave similarly under the following chromatographic conditions, their concentrations were calculated based on the calibration curve plotted for 6-gingerol, a primary compound representative of the Zingiberaceae family. The method was validated for quantitative determinations as previously described [29]. Briefly, based on the obtained determinations, the calibration curve was plotted, which was characterized by the following equation: y = 5,439,294.167x + 83,057.91667 (R^2^ = 0.999). The repeatability of the quantitative determinations was higher than 90% and the recovery determined for the standard was equal to 95%. The limit of detection (LOD) value for the investigated standard in the prepared method was calculated to be 0.34 mg/kg, whereas the limit of quantification (LOQ) was 1.12 mg/kg.

### 4.8. Bioaccessibility calculation

The bioaccessibility of each compound was calculated according to a previously described procedure [29,31] based on the quantitative determinations using the HPLC-DAD method and the following formula:

$$B=\left(\frac{Ia-Ib}{Ic}\right)\times 100\%$$

where *Ia*—content after digestion, *Ib*—content after digestion and filtration, and *Ic*—initial content in the extract (before digestion).

### 4.9. Statistical Analysis

The results were reported as means and standard deviations. The Shapiro–Wilk test was used to verify normal distribution for the analyzed variables. To examine the diversity of variance in the four independent groups and to determine which differences between them are statistically significant, univariate analysis of variance (ANOVA) and post hoc Tukey tests were used. All statistical analyses were performed using the statistical software package PS IMAGO PRO (IBM SPSS Statistics 29). The value of *p* < 0.05 was considered significant.

## 5. Conclusions

In the present study, the bioaccessibility of six active components in the root of *Alpinia officinarum* was evaluated: pinobanksin, apigenin, galangin, kaempferide, acacetin, and chrysin. The digestion process employing various food matrices significantly influenced the bioaccessibility of the analyzed compounds. Galangin emerged as the predominant compound in the root and exhibited the highest bioaccessibility across all diet types. The variability in bioaccessibility among the individual compounds underscores the substantial impact of the food matrix on their bioavailability. However, it is challenging to identify a single dietary model that optimally enhances the bioaccessibility of all studied compounds. Notably, except kaempferide, the presence of food generally increased the bioaccessibility of the active compounds, suggesting that dietary nutrients may provide a protective effect against oxidative degradation. The food matrix likely shields these substances from the detrimental effects of digestive juices, particularly in the context of a high-fiber diet that is rich in dietary fiber and antioxidants such as vitamin C, carotenoids, and polyphenols.

The observed variability in results can be attributed to the unique physicochemical properties of each compound and their specific interactions with dietary components. This indicates that the influence of food on the bioaccessibility of individual compounds within *Alpinia officinarum* is compound-specific. Based on the findings, it can be concluded that no singular dietary model is universally beneficial for enhancing the bioaccessibility of the compounds present in *Alpinia* root. Nonetheless, the results reveal a positive impact of the food matrix on the availability of these compounds, potentially enhancing their biological significance for the organism.

## Figures and Tables

**Table 1:** Tentatively identified compounds in the *A. officinarum* extract in negative ion mode.

No.	Ion (+/−)	Retention Time	Molecular Formula	*m*/*z* Calculated	*m*/*z* Experimental	Delta (ppm)	RDB	MS/MS Fragments	Proposed Compound
1	-	10.0	C_11_H_14_O_3_	193.087	193.087	0.09	5	193.0871 179.0871	Zingerone
2	-	12.3	C_9_H_10_O_2_	149.0608	149.0609	−0.65	5	107.9953 105.0566	3-Phenylpropanoic acid
3	-	15.0	C_15_H_12_O_5_	271.0612	271.0612	−0.01	10	151.0036 119.0503	Pinobanksin
4	-	16.3	C_15_H_10_O_6_	285.0405	285.0413	−2.93	11	239.0352 213.0548	Kaempferol
5	-	18.1	C_10_H_18_O	153.1285	153.129	−3.32	2	113.0275	1,8-Cineole
6	-	21.5	C_15_H_10_O_4_	253.0506	253.0513	−2.63	11	209.0603 181.0651	Chrysin
7	-	21.7	C_16_H_12_O_7_	315.051	315.0509	0.4	11	283.0240 255.0278	Isorhamnetin
8	-	22.4	C_15_H_12_O_4_	255.0663	255.0665	−0.85	10	213.0558 151.0030	Pinocembrin
9	-	22.8	C_15_H_10_O_5_	269.0455	269.0459	−1.3	11	227.0360 223.0411 213.0564	Galangin
10	-	23.1	C_16_H_12_O_6_	299.0561	299.0558	1.04	11	284.0318 164.0104	Kempferide
11	-	23.5	C_15_H_10_O_5_	269.0455	269.0459	−1.31	11	227.0343 213.0533	Apigenin
12	-	24.0	C_16_H_12_O_5_	283.0612	283.0611	0.34	11	268.0382 239.0352 211.0402	Acacetin

**Table 2:** Content of active substances in the root of *A. officinarum*.

	Pinobanksin (n = 6) X ± SD	Apigenin (n = 6) X ± SD	Galangin (n = 6) X ± SD	Kaempferide (n = 6) X ± SD	Acacetin (n = 6) X ± SD	Chrysin (n = 6) X ± SD
Content [mg/kg] in the powdered root	42.0 ± 5.4	140.0 ± 10.1	1090.9 ± 102.5	173.8 ± 14.9	96.1 ± 9.5	98.8 ± 7.8

X—mean, SD—standard deviation.

**Table 3:** Bioaccessibility [%] of active substances in the control (only water, no nutrients) and studied samples. The means sharing at least one different letter in a row differ significantly at *p* ≤ 0.05. Means without any shared letters do not show significant differences.

	Control Sample (n = 6) X ± SD	High-Fiber Diet (n = 6) X ± SD	Basic Diet (n = 6) X ± SD	Standard Diet (n = 6) X ± SD	ANOVA *p*
Pinobanksin	7.19 ± 0.70 ^(b,c,d)^	2.30 ± 0.70 ^(a,c)^	16.55 ± 2.44 ^(a,b,d)^	3.40 ± 0.69 ^(a,c)^	<0.0001
Apigenin	5.56 ± 0.51 ^(b,d)^	20.57 ± 2.44 ^(a,c,d)^	5.88 ± 0.93 ^(b,d)^	40.72 ± 2.94 ^(a,b,c)^	<0.0001
Galangin	17.36 ± 0.81^(c,d)^	19.17 ± 1.55 ^(c,d)^	23.64 ± 2.32 ^(a,b,d)^	36.13 ± 3.69 ^(a,b,c)^	<0.0001
Kaempferide	8.90 ± 0.32 ^(b,c,d)^	6.23 ± 0.44 ^(a,c,d)^	3.44 ± 0.20 ^(a,b,d)^	5.06 ± 0.38 ^(a,b,c)^	<0.0001
Acacetin	29.18 ± 3.03	25.81 ± 3.30 ^(b)^	30.19 ± 2.08 ^(c)^	27.53 ± 0.37	0.0309
Chrysin	15.12 ± 1.92 ^(b,d)^	63.6 ± 3.21 ^(a,c,d)^	17.08 ± 2.00 ^(b,d)^	3.33 ± 0.76 ^(a,b,c)^	<0.0001

X—mean; SD—standard deviation.

**Table 4:** Solvent system used in the LC-MS method.

Flow [mL/min]	Analysis Time [min]	Solvent A [%]	Solvent B [%]
0.2	0	99	1
	3	80	20
	30	25	75
	36	5	95
	38	5	95
	38.5	80	20
Detection UV [nm]		210, 230, 254, 280, 290, 320	

## Data Availability

The original contributions presented in this study are included in the article/Supplementary Material. Further inquiries can be directed to the corresponding author.

## Supplementary Materials

The following supporting information can be downloaded at: https://www.mdpi.com/article/10.3390/molecules30224429/s1, Figure S1: Bioaccessibility [%] of active substances in the control (only water, no nutrients) and studied samples; Table S1: Composition of individual diets; Table S2: Nutritional values of diets; Table S3: The MS/MS spectra of the tentatively identified components.
