# Supplementary material for: Influence of Diet on the Bioaccessibility of Active Substances from Alpinia officinarum Using In Vitro Digestion Model
Source: Molecules. 2025 Nov 16;30(22):4429. doi: 10.3390/molecules30224429 (PMC12655224; doi:10.3390/molecules30224429)

SUPPLEMENTARY FILE

Influence of diet on the bioaccessibility of active substances from *Alpinia officinarum* using in vitro digestion model

Wojciech Koch [1,*], Justyna Zagórska [1], Agnieszka Jaworowska [1,], Paweł Jagielski [2], Ewa Bartusiak-Szcześniak [3] and Wirginia Kukula-Koch [4]

[1] Department of Food and Nutrition, Medical University of Lublin, 4a Chodzki Str., 20-093 Lublin, Poland: justyna.zagorska@umlub.edu.pl; agnieszka.jaworowska@umlub.edu.pl
[2] Department of Nutrition and Drug Research, Institute of Public Health, Faculty of Health Sciences, Jagiellonian University Medical College, 31-066 Kraków, Poland; paweljan.jagielski@uj.edu.pl
3 Department of Pharmaceutical Sciences, Collegium Medicum, Jan Kochanowski University in Kielce, IX Wieków Kielc 19a, 25-516 Kielce, Poland; ewa.bartusiak-szczesniak@ujk.edu.pl
[4] Department of Pharmacognosy with Medical Plants Garden, Medical University of Lublin, 1 Chodzki Str., 20-093 Lublin, Poland; virginia.kukula@gmail.com

* Correspondence: kochw@interia.pl; Tel.: +48-81-448-7143

Table S1. Composition of individual diets [30].

	**High-fiber diet**		**Basic diet**		**Standard diet**	
**Breakfast**	milk<br>oat flakes or others<br>wholemeal bread<br>cottage cheese<br>jam	400 mL<br>50 g<br>80 g<br>100 g<br>25 g	cereal coffee + milk<br>white bread<br>butter<br>cottage cheese<br>(+ 10 g chives and 25 g cucumber)	250 mL<br>80 g<br>10 g<br>100 g	hot chocolate<br>mixed bread<br>ham sausages<br>mustard	250 mL<br>80 g<br>150 g<br>10 g
**Lunch**	tea<br>mixed bread<br>rennet cheese<br>ham<br>tomato<br>banana	250 mL<br>80 g<br>50 g<br>40 g<br>150 g<br>120 g	tea<br>mixed bread<br>chicken pate<br>apple	250 mL<br>60 g<br>120 g<br>150 g	coffee with milk<br>crispbread<br>gouda cheese	120 mL<br>20 g<br>50 g
**Dinner**	strawberry compote<br>red borscht (30g beetroot + 25 g vegetables: carrot, parsley, celery)<br>potatoes<br>pork chop	200 mL<br>400 mL<br>300 g<br>150 g<br>300 g	strawberry compote with fruit pieces (30 g)<br>vegetable soup (100 g vegetables: carrot, celery, parsley, cauliflower)<br>potatoes<br>chicken cutlet	250 mL<br>400 mL<br>300 g<br>150 g	compote<br>coffee with milk<br>tomato soup with noodles<br>potatoes<br>grilled cod<br>sauerkraut salad	220 mL<br>120 mL<br>400 mL<br>300 g<br>200 g<br>120 g<br>25 g

	cooked vegetables (carrot + peas (1:1) with butter)<br>apple	150 g	red cabbage salad	120 g	milk chocolate with nuts	
**Supper**	hot chocolate<br>mixed bread<br>butter<br>chicken ham<br>pepper	220 mL<br>80 g<br>10 g<br>80 g<br>150 g	tea with milk<br>meatballs in sauce<br>noodles<br>salad (carrot, apple, mayonnaise)<br>yeast cake with crumble	250 mL<br>100 g<br>120 g<br>100 g<br>50 g	tea<br>mixed bread<br>sausages<br>pickled cucumber	250 mL<br>60 g<br>80 g<br>70 g

Table S2. Nutritional values of diets [30].

	**High-fiber diet**	**Basic diet**	**Standard diet**
**Protein**	151 g (19% of energy)	104 g (16.4% of energy)	138.4 g (22.6% of energy)
**Fat**	120.3 g (34% of energy)	101 g (35.6% of energy)	114.4 g (42% of energy)
**Carbohydrates**	423.2 g (47% of energy)	331 g (48.1% of energy)	237.4 g (35.4% of energy)
**Fiber**	50.3 g	27.8 g	21.0 g
**Energy**	3186.6 kcal	2545 kcal	2451 kcal
**Vitamin A**	4.75 mg	2.83 mg	0.47 mg
**Vitamin C**	307.2 mg	76.6 mg	50.7 mg
**Vitamin E**	24.8 mg	15.7 mg	14.3 mg
**Calcium**	1618.5 mg	502 mg	1067 mg
**Sodium**	3410.8 mg	2207 mg	4053 mg
**Potassium**	6924.8 mg	4700 mg	5156 mg
**Magnesium**	689.3 mg	349 mg	497 mg
**Iron**	21.4 mg	13.6 mg	15 mg

The nutritional value of the diets was calculated using Dietetyk 2006 software (Jumar, Poland).

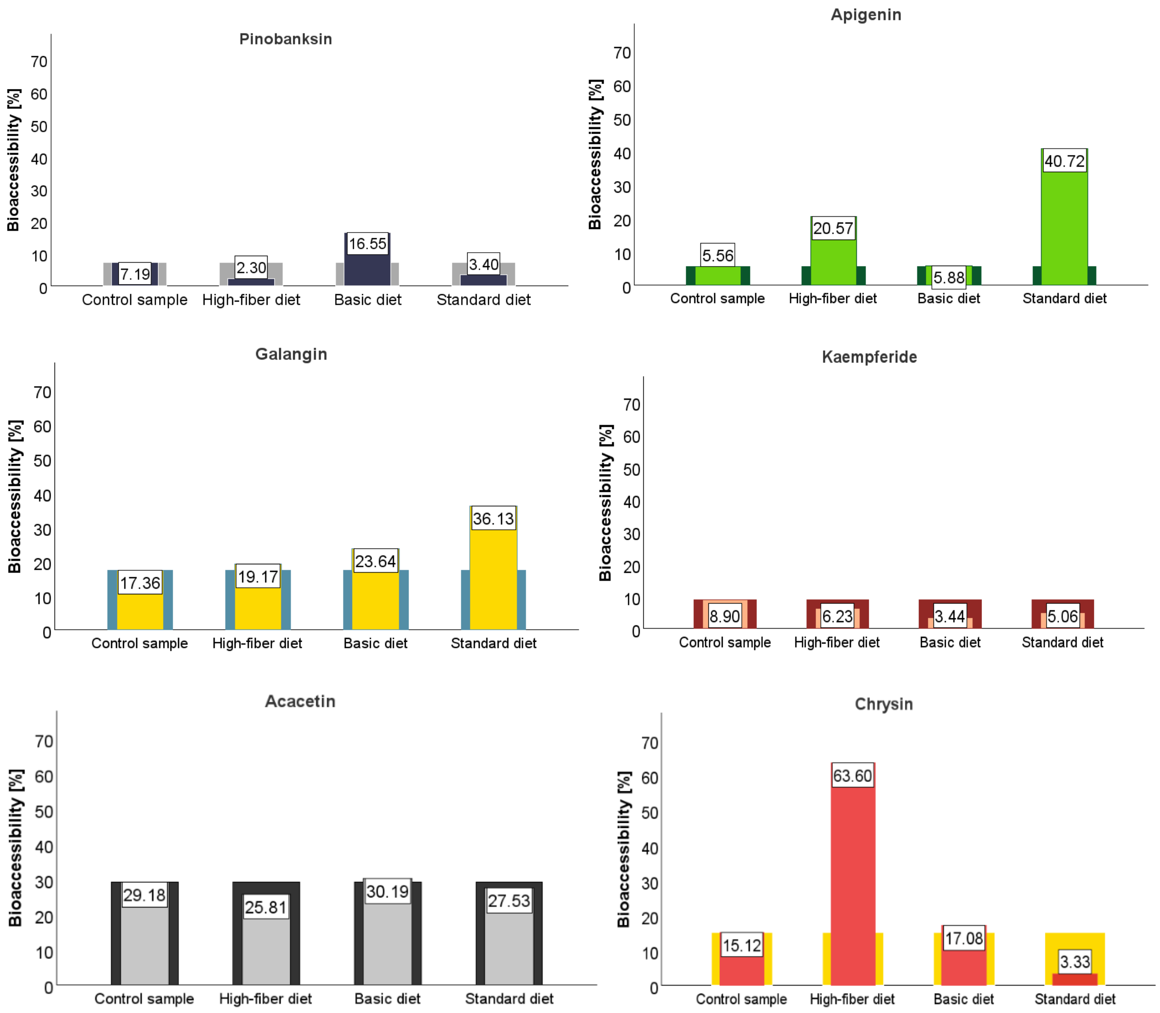

Figure S1. Bioaccessibility [%] of active substances in the control (only water, no nutrients) and studied samples. An up or down arrow indicates a statistically significant difference with respect to the control sample ($p \leq 0.05$).

Table S3. The MS/MS spectra of the tentatively identified components.

No.	Proposed Compound	
1	Zingerone	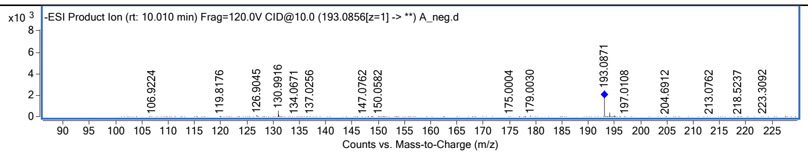

2	3-Phenylpropanoic acid	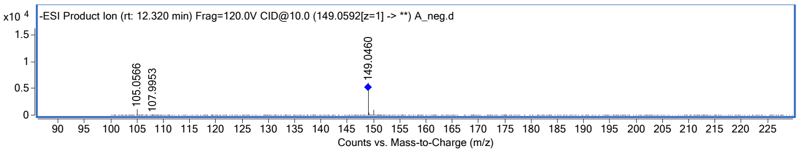
3	Pinobanksin	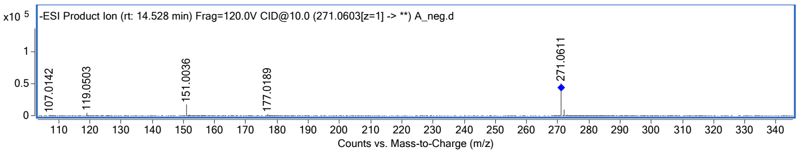
4	Kaempferol	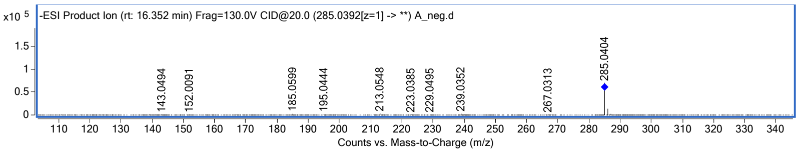
5	1,8-Cineole	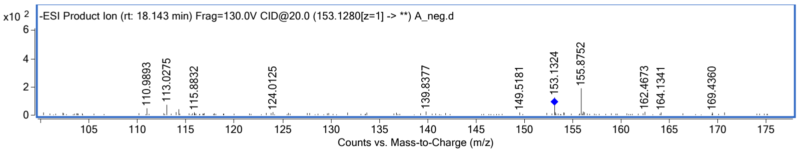
6	Chrysin	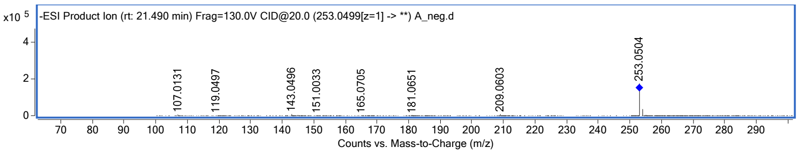
7	Isorhamnetin	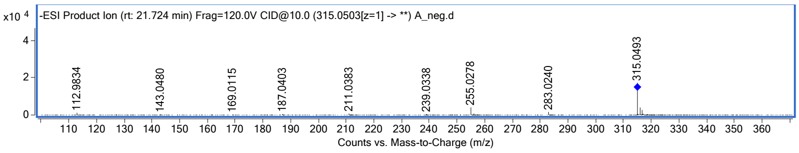
8	Pinocembrin	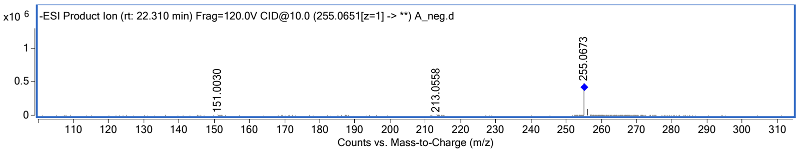

9 Galangin

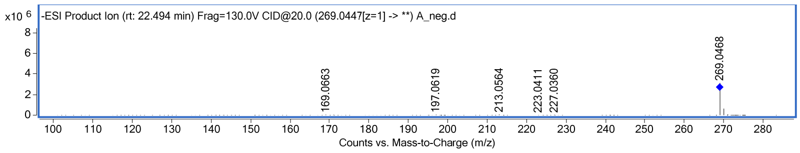

10 Kempferide

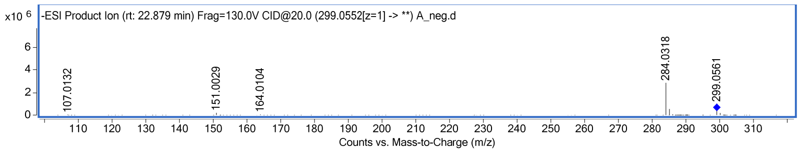

11 Apigenin

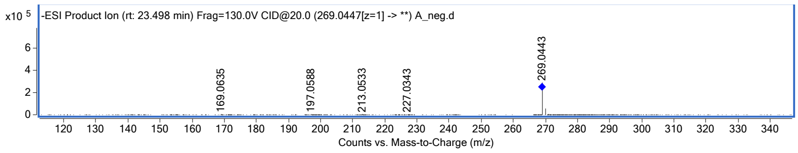

12 Acacetin

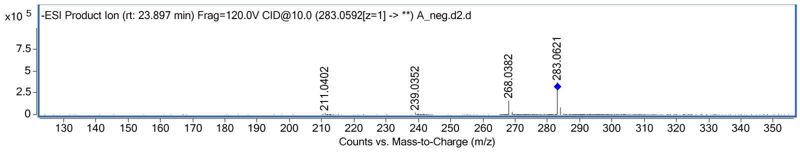

Supplement: Supplementary file 1 [file molecules-30-04429-s001.zip › molecules-3937416-supplementary.pdf]
